# Green Synthesis, Optimization, and Characterization of CuO Nanoparticles Using *Tithonia diversifolia* Leaf Extract

**DOI:** 10.3390/nano15151203

**Published:** 2025-08-06

**Authors:** S. S. Millavithanachchi, M. D. K. M. Gunasena, G. D. C. P. Galpaya, H. V. V. Priyadarshana, S. V. A. A. Indupama, D. K. A. Induranga, W. A. C. N. Kariyawasam, D. V. S. Kaluthanthri, K. R. Koswattage

**Affiliations:** 1 Department of Biosystems Technology, Faculty of Technology, Sabaragamuwa University of Sri Lanka, Belihuloya 70140, Sri Lanka; sahani@tech.sab.ac.lk; 2 Faculty of Graduate Studies, Sabaragamuwa University of Sri Lanka, Belihuloya 70140, Sri Lanka; vimukkthi@tech.sab.ac.lk (H.V.V.P.); nipunichanika96@gmail.com (W.A.C.N.K.); 3 Center for Nanodevice Fabrication and Characterization, Faculty of Technology, Sabaragamuwa University of Sri Lanka, Belihuloya 70140, Sri Lanka; chanakagalpaya@gmail.com (G.D.C.P.G.); amalka@tech.sab.ac.lk (S.V.A.A.I.); ashaninduranga@tech.sab.ac.lk (D.K.A.I.); 4 Department of Engineering Technology, Faculty of Technology, Sabaragamuwa University of Sri Lanka, Belihuloya 70140, Sri Lanka; 5 Department of Plant and Molecular Biology, Faculty of Science, University of Kelaniya, Kelaniya 11600, Sri Lanka; dvska211@kln.ac.lk

**Keywords:** characterization, green synthesis, CuO nanoparticles, *Tithonia diversifolia*

## Abstract

Green synthesis of copper oxide (CuO) nanoparticles offers a sustainable alternative to conventional chemical methods that often involve toxic reagents and harsh conditions. This study investigates the use of *Tithonia diversifolia*, an invasive species in Sri Lanka, as a bioreductant for the eco-friendly fabrication of CuO nanoparticles. Using copper sulfate (CuSO_4_·5H_2_O) as a precursor, eight treatments were conducted by varying precursor concentration, temperature, and reaction time to determine optimal conditions. A visible color change in the reaction mixture initially indicated nanoparticle formation. Among all the conditions, treatment T4 (5 mM CuSO_4_, 80 °C, 2 h) yielded the most favorable results in terms of stability, morphology, and crystallinity. UV-Vis spectroscopic analysis confirmed the synthesis, with absorbance peaks between 265 and 285 nm. FTIR analysis revealed organic functional groups and characteristic metal–oxygen vibrations in the fingerprint region (500–650 cm^−1^), confirming formation. SEM imaging showed that particles were mainly spherical to polygonal, averaging 125–150 nm. However, dynamic light scattering showed larger diameters (~240 nm) due to surface capping agents. Zeta potential values ranged from −16.0 to −28.0 mV, indicating stability. XRD data revealed partial crystallinity with CuO-specific peaks. These findings support the potential of *T. diversifolia* in green nanoparticle synthesis, suggesting a low-cost, eco-conscious strategy for future applications.

## 1. Introduction

The history of nanotechnology can be traced back over 4500 years; however, its modern development began approximately five decades ago. Since then, it has undergone substantial advancements across different disciplines. Nanoparticles are typically defined as particles with dimensions of ≤100 nm. Nanotechnology has multidisciplinary applications across biology, chemistry, physics, engineering, medicine, automobile, tissue culture, and more [1,2,3,4,5]. In recent decades, nanotechnology-based research efforts have led to the development and commercialization of synthesized products [6,7,8]. Production of nanoparticles can be achieved via two fundamental approaches: the top-down method, where larger particles are broken down into smaller ones, and the bottom-up method, where nanoparticles are built from atoms or molecules. Based on these two approaches, three methods are commonly employed to produce nanoparticles: physical, chemical, and mechanical [3].

Physical methods, such as laser ablation (top-down), physical vapor deposition (bottom-up), and plasma synthesis (bottom-up) use heat, pressure, plasma, and lasers to synthesize nanoparticles. Chemical methods, such as sol-gel, hydrothermal, coprecipitation, and chemical vapor deposition, rely on chemical reactions to produce nanoparticles using the bottom-up approach. Mechanical methods, inherently top-down, use machines to grind or crush materials into nanoparticles, such as ball milling, cryo-milling, and mechanical alloying [6,7].

However, these three methods often involve harsh conditions and the use of toxic and hazardous chemicals, leading to the release of harmful by-products and contributing to environmentally unsustainable practices [9]. To address these challenges, the concept of green synthesis has emerged, utilizing microorganisms and plants as eco-friendly alternatives. Microorganisms produce different secondary metabolites and biomolecules, such as enzymes and proteins, while plants are rich in phytochemicals. These biological compounds can act as reducing agents, stabilizers, and capping agents in the synthesis of nanoparticles [8].

The typical plant-based nanoparticle synthesis process begins with the selection of a microorganism or a specific plant species and its appropriate part (e.g., leaf, stem, or root). This plant material is crushed to obtain an aqueous or solvent-based extract, which is subsequently filtered or centrifuged to remove impurities. The clarified plant extract is then mixed with a precursor solution, usually a metal salt, such as silver nitrate (AgNO_3_) or copper sulfate (CuSO_4_). Reaction conditions, particularly pH, temperature, and continuous stirring, must be carefully controlled to ensure the production of nanoparticles with uniform size and shape. A visible color change in the reaction mixture is often considered a preliminary indication of nanoparticle formation due to surface plasmon resonance effects [10,11,12]. The overall process of biogenic nanoparticle synthesis, or bionanosynthesis, generally occurs in three major stages: activation, growth, and stabilization. In the activation phase, the metal ions (e.g., Ag^+^, Cu^2+^) are reduced to their metallic state (Ag^0^, Cu^0^) by bioactive compounds present in the extract, such as flavonoids, terpenoids, phenolics, proteins, and reducing sugars. These atoms then begin to form small clusters, in a process called nucleation. In the growth phase, these seeds join together to form larger nanoparticles with defined shapes and sizes. In the final stage, stabilization, molecules in the extract bind to the surface of the nanoparticles, preventing them from clumping together and keeping them stable in solution [13]. The success of the synthesis is typically confirmed through visual observation of a color change and further verified using high-throughput techniques.

Conventional synthesis processes typically utilize noble metals such as silver (Ag), gold (Au), and titanium. However, copper (Cu) is considered a promising, low-cost alternative. In this study, *Tithonia diversifolia* was used for the green synthesis of nanoparticles. *T. devrsifolia*, commonly known as Mexican sunflower or wild sunflower, is native to Mexico and Central America. It has since been introduced to other continents, where it has become an aggressive invasive species [14]. Despite their invasive nature, the plant has been utilized in traditional medicine since ancient times to treat various diseases, including stomach disorders and skin infections, both topically and orally. According to previous studies, the leaves of *T. diversifolia* contain a wide variety of bioactive compounds with antibacterial, antioxidant, and therapeutic properties, including terpenoids, alkaloids, flavonoids, phenolics, and saponins [14,15,16]. Owing to these biological attributes, *T. diversifolia* is well-suited to function as a reducing, stabilizing, and capping agent in the green synthesis of nanoparticles. Harnessing the phytochemical potential of this widely distributed invasive plant offers a cost-effective and environmentally sustainable approach to nanoparticle production while also contributing to ecological efforts aimed at managing invasive species. While some studies have employed *T. diversifolia* extracts for nanoparticle synthesis, few have systematically optimized key synthesis parameters, such as precursor concentration, temperature, and reaction time, or conducted a comprehensive physicochemical characterization of the resulting CuO nanoparticles [17]. The present study addresses this gap by developing an optimized, eco-friendly method for CuO nanoparticle synthesis using *T. diversifolia* leaf extract and characterizing the nanoparticles across multiple dimensions. The purpose of this study is to develop an optimized, eco-friendly method for CuO nanoparticle synthesis using *T. diversifolia* leaf extract, with the aim of producing quality nanoparticles for potential use in future biotechnological applications (incorporating synthesized nanoparticles into the optimization of plant tissue culture protocols).

## 2. Materials and Methods

Commercially available high-purity copper (II) sulfate pentahydrate (CuSO_4_·5H_2_O) was used for all experiments. Aqueous solutions were prepared by dissolving the salt in distilled water. Fresh leaves of *T. diversifolia* were collected and used as the biological component in the synthesis procedure.

### 2.1. Preparation of T. diversifolia Leaf Extract

A voucher specimen was prepared from the collected plant sample and submitted to the Royal Botanic Gardens, Peradeniya, for authentication. Then, the leaves of *T. diversifolia* were collected and washed thoroughly under running tap water to remove dust and other surface contaminants. The leaves were air-dried for 24 h and subsequently oven-dried at 40 °C for two days. Once dried, the leaves were ground into a fine powder using a grinder and passed through a mesh sieve (60–300 µm) to ensure uniform particle size. The resulting powder was stored at 4 °C for further use. For the phytochemical extraction, 20 g of leaf powder was mixed with 200 mL of distilled water and heated at 70 °C for two hours under continuous stirring (DLAB SCIENTIFIC CO., LTD MS-H380 Pro Digital Magnetic Hot Plate Stirrer, Beijing, China). The extract was then filtered using Whatman No.1 filter paper with a Buchner funnel, and the filtrate was used for the nanoparticle synthesis process.

### 2.2. Synthesis of CuO Nanoparticles (CuO NPs)

**Figure 1:**
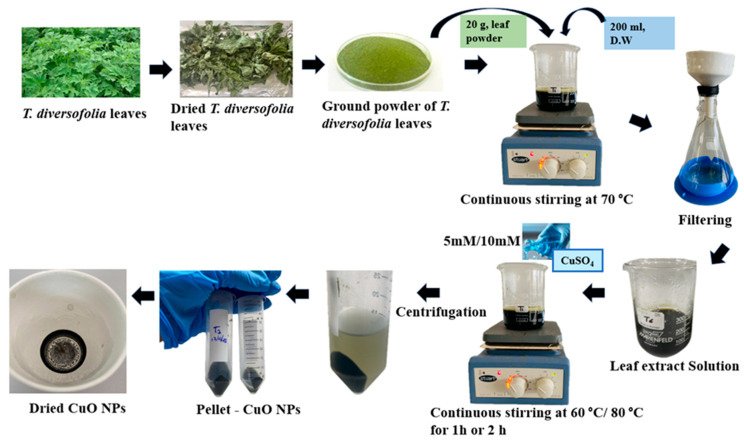
Schematic representation of *T. diversifolia* leaf as a source of green synthesis of CuO NPs.

Two concentrations of copper (II) sulfate pentahydrate (CuSO_4_·5H_2_O)—5 mM and 10 mM—were prepared by dissolving the respective amounts in distilled water. To optimize CuONPs synthesis, eight experimental treatments (T1–T8) were conducted by varying CuSO_4_ concentration (5 mM and 10 mM), reaction temperature (60 °C and 80 °C), and stirring time (1 h and 2 h). In each treatment, 200 mL of *T. diversifolia* leaf extract was mixed with 200 mL of CuSO_4_·5H_2_O solution (either 5 mM or 10 mM) and stirred continuously at 400 rpm. The reaction conditions were varied, with temperatures set at either 60 °C or 80 °C and reaction durations of 1 h or 2 h, as detailed in Table 1. After the synthesis of CuO nanoparticles, the reaction mixtures were centrifuged (Hermle Z36 HK Super Speed Centrifuge, Sayreville, NJ, USA) at 15,650× *g* (10,000 rpm) for 15 min to separate the nanoparticles from the supernatant. The resulting pellet was then washed three times with distilled water, each wash followed by centrifugation under the same conditions. Finally, the synthesized nanoparticles were oven-dried at 60 °C for four hours (Figure 1).

### 2.3. Characterization of Synthesized CuO NPs

The Scanning Electron Microscope (SEM) (ZEISS EVO 15, Jena, Germany) with a magnification of 5.00K× was used for morphological and structural analyses. X-ray diffraction (XRD) (Bruker D8 Advance ECO, Karlsruhe, Germany) was used for the structural information of CuO NPs. Particle size distribution and zeta potential were measured using a dynamic light scattering (DLS) analyzer (Anton Paar, Litesizer 500, Graz, Austria). Infrared (IR) spectra were obtained using a Fourier-Transform Infrared (FTIR) spectrometer (Thermo Scientific Nicolet iS10, Waltham, MA, USA) equipped with an ITX diamond attenuated total reflectance (ATR) accessory, scanning the spectral range of 400–4500 cm^−1^. The UV–Visible absorption spectra were recorded using a UV–Vis spectrophotometer (Shimadzu UV-2600i/UV-2700i Plus, Kyoto, Japan) over a wavelength range of 200–800 nm.

### 2.4. Phytochemical Screening of Leaf Extract

Phytochemical extraction was carried out using the Soxhlet extraction method. A 15.0 g sample of dried leaf powder was extracted with 150.0 mL of absolute ethanol for 4 h at 50 °C. The excess solvent was evaporated using a rotary evaporator at 40 °C, and the crude extract was redissolved in absolute ethanol, then passed through a sodium sulfate column to remove any residual water and filtered through a microfilter before being subjected to gas chromatography mass spectrometry (GC-MS).

The initial oven temperature was maintained at 50 °C. Helium was used as the carrier gas at a constant flow rate of 1 mL/min, and an HP-5MSUI capillary column (30 m × 0.250 mm × 0.25 μm) was used as the stationary phase. Following standard procedures, 1 μL of the sample was injected into the GC-MS (Agilent 5977B GC/MSD, Santa Clara, CA, USA). The oven temperature program began at 100 °C, increased to 110 °C at a rate of 10 °C/min, and then ramped to 280 °C at a rate of 5 °C/min, which was held for 9 min. The total run time was 49 min. The flow was split at a ratio of 10:1 before introduction into the MS detector. Mass spectral data were acquired using an MS detector (Agilent 8890 GC system, Santa Clara, CA, USA) in electron impact (EI) scan mode at 70 eV, with a mass scan range of 40–550 *m*/*z*. Spectral data were obtained using the MSD Enhanced ChemStation F.01.03.2357 provided by Agilent, Santa Clara, CA, USA. The resulting spectra were compared with entries in the NIST/EPA/NIH Mass Spectral Library using the Automatic Mass Spectral Deconvolution and Identification System (AMDIS) program and the NIST Mass Spectra Search program.

## 3. Results

The synthesis of CuO nanoparticles has been reported to be influenced by several parameters, including precursor concentration, reaction temperature, and stirring time [18]. In this study, the focus was placed on optimizing precursor concentration, temperature, and stirring time. The selection of these specific parameter ranges was guided by the established literature [8,19] and adapted from the experimental design reported by [20]. Two different temperatures were selected to examine the thermal effect on nucleation and crystal growth. By varying the copper precursor concentration and reaction time, conditions were established that yielded CuO nanoparticles with improved crystallinity, dispersion stability, and morphology.

### 3.1. SEM Analysis

**Figure 2:**
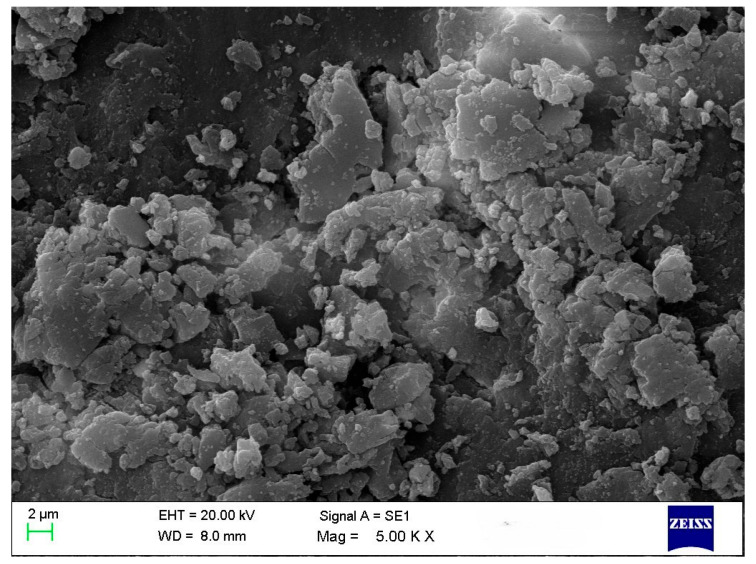
SEM image of CuO NPs in T4.

A common technique is used to determine the average particle size and analyze the morphology of materials. All treatments in this study revealed the presence of polygonal and spherical nanoparticles with some aggregation (~125–150 nm) (Figure 2). According to the literature, nanoparticle morphology and size vary depending on the biological samples used. Ref. [8] reported circular nanoparticles with rough surface clusters ranging from 10 to 60 nm. Ref. [21] observed spherical and cuboidal shapes with agglomeration, while [22] found spherical particles approximately 20 nm in size. Ref. [23] noted that due to the high surface-area-to-volume ratio, nanoparticles possess high surface energy and tend to agglomerate to minimize it; this agglomeration is primarily caused by van der Waals forces, with reported sizes reaching 1.48 µm. Ref. [24] also observed spherical and hexagonal nanoparticles, including some with large sizes attributed to agglomeration.

### 3.2. XRD Analysis

The XRD technique is used to determine the crystallinity of the synthesized nanoparticles. The results revealed an amorphous nature in the nanoparticles, indicated by the broadness and absence of sharp diffraction peaks [25]. Although strong peaks were not observed, distinguishable peaks were present at 2θ values of 52.957° (T1), 32.246° (T3), 38.162°, 58.545° (T4), and 32.245° (T8). According to the literature, these 2θ values correspond to crystallographic planes (020, 110, 111, 202, and 110, respectively) based on data from the International Centre for Diffraction Data [8,19,26]. These observations confirm the formation of CuO nanoparticles.

Crystallite sizes calculated using the Debye–Scherrer equation d = Kλ/βcosθ (where K = 0.89, λ = 0.154 nm (X-ray wavelength), β is the full width at half maximum (FWHM, radian), and θ is the diffraction angle) revealed average particle sizes of 36.9 (T1), 61.5 (T3), 42.2 (T4), 90.1 (T4), and 39.1 (T8) nm.

**Figure 3:**
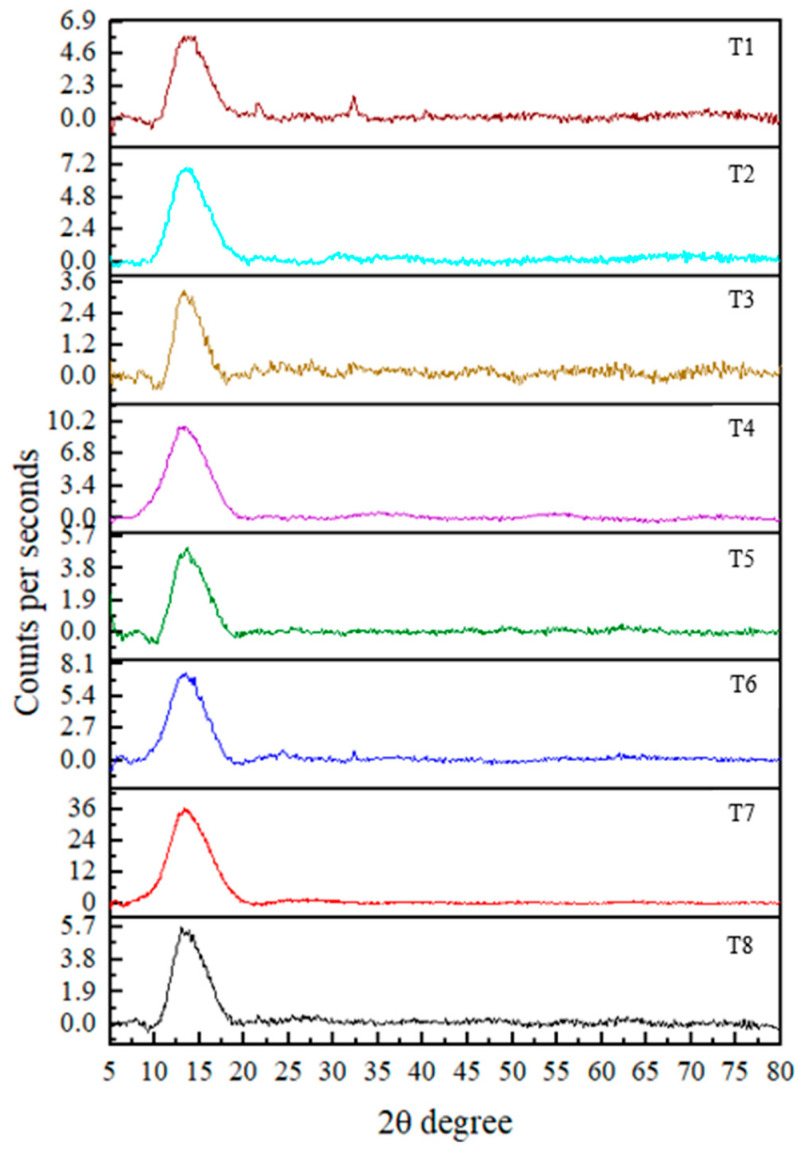
XRD pattern for CuO NPs in each treatment.

Notably, all treatments showed diffraction peaks within the 10–20° range, suggesting the presence of residual plant material associated with the nanoparticles. This may have contributed to variations in the results. Ref. [27] reported peaks at 18.3° and 24.4°, which were also indicative of CuO nanoparticle formation. However, the overall diffraction pattern in the current study suggests that the nanoparticles are not in a fully pure phase. In addition, no reference data were found in the literature corresponding to certain 2θ values such as 55°, 63°, 42°, 62°, 40°, 69°, and 63°, which were observed in the current study in CuO NPs. This suggests the presence of unidentified phases or impurities in the synthesized nanoparticles (Figure 3). Finally, the findings suggest that further purification (such as additional washing, calcination, or optimization of synthesis parameters) of the nanoparticles is necessary to improve crystallinity and remove residual organic matter. Implementing such strategies in future studies would likely enhance the crystallinity and purity of the nanoparticles, increasing their suitability for applications requiring high material quality.

### 3.3. Dynamic Light Scattering Analysis

#### 3.3.1. Particle Size

Typically, nanoparticles are described as having sizes between 1 and 100 nm. However, compared to values reported in earlier investigations [28,29], the nanoparticles synthesized in this study exhibited significantly larger sizes (Table 2). This suggests that the particles may not fall within the nanoscale range. It should be emphasized that DLS was used to estimate particle size; this technique measures the hydrodynamic diameter, which includes both the nanoparticle core and any surrounding organic or solvent layers. Therefore, the apparent increase in particle size may result from both the metallic core and the biomolecular capping agents used in the green synthesis process.

The larger particle sizes observed can be attributed to several factors, including particle aggregation due to insufficient stabilization, slow nucleation rates, and the presence of high levels of phytochemicals that act as both capping and reducing agents. Phytochemicals from the plant extract may adhere to the nanoparticle surface and form a shell, thereby increasing the overall measured diameter. Additionally, the literature indicates that reaction temperature can influence nanoparticle size. Higher temperatures may accelerate nucleation and produce smaller particles [30], although other studies suggest that, depending on reaction kinetics and extract composition, elevated temperatures can also lead to larger particle sizes [31,32]. However, treatments T1, T3, T4, and T8 recorded smaller particle sizes than the other treatments (Table 2). In this study, dispersion-enhancing procedures such as sonication or sample dilution were not applied prior to DLS analysis. Incorporating such methods in future work may improve the accuracy and reliability of hydrodynamic size measurements by minimizing aggregation and ensuring better dispersion.

According to previous studies, the PDI ranges from 0.01 for monodispersed particles to 0.5–0.7 for samples with broader size distributions. A PDI value greater than 0.7 typically indicates a very broad distribution and poor nanoparticle stability [33,34]. In this study, all treatments showed PDI values between 0.222 and 0.473, which are within the acceptable range for green-synthesized nanoparticles. Ref. [26] reported a PDI of 0.288 in a similar plant-based CuO nanoparticle synthesis, further supporting that moderate PDI values are commonly observed and still represent successful nanoparticle formation. Therefore, the polydispersity indices observed in our treatments confirm the formation of nanoparticles with acceptable size distributions under green synthesis conditions.

#### 3.3.2. Zeta Potential

**Figure 4:**
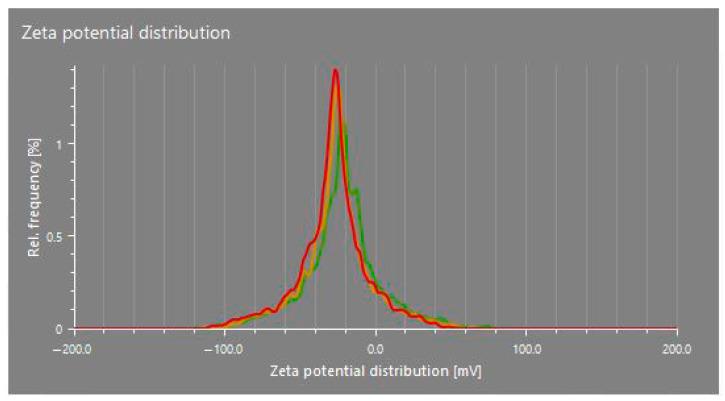
Zeta potential distribution of CuO NPs in T4. Different colors represent the zeta potential distribution of different replicates.

The zeta potential values of the CuO nanoparticles synthesized in this study (dispersed in distilled water) ranged from −16.0 mV to −28.0 mV across different treatments, indicating a generally stable colloidal system (Table 3; Figure 4). These relatively high negative values suggest that the nanoparticles possess sufficient surface charge to repel each other, thereby preventing aggregation and contributing to the moderate stability of the nanoparticle suspension. Zeta potential reflects the surface charge of nanoparticles and is influenced by the chemical composition of the capping agents. In this study, the phytochemicals present in the plant extracts likely played a key role in stabilizing the CuO nanoparticles by acting as both reducing and capping agents.

These findings are consistent with earlier research, supporting both the proposed mechanisms and the observed zeta potential values [28,35]. However, ref. [28] reported a zeta potential of −3.50 mV in their study, which enhanced the stability of CuO nanoparticles by inhibiting aggregation over an extended period. The higher negative zeta potential observed in some treatments, particularly −28.0 mV for T8, suggests a greater degree of electrostatic stabilization, possibly due to a denser or more effective phytochemical-based capping layer provided by the specific plant extract used in that treatment.

### 3.4. FTIR Analysis

The FTIR spectrum provides important information about the bonds present in the samples. When infrared light passes through a sample, molecules absorb energy and vibrate, creating characteristic peaks in the spectrum. The FTIR spectrum is generally divided into two regions: the functional group region (1500–4000 cm^−1^) and the fingerprint region (400–1500 cm^−1^). The synthesized CuO nanoparticles (CuO NPs) in this study exhibited various vibrational peaks, possibly due to the capping of phytochemicals around the nanoparticles [8,23]. In addition to organic functional groups, metal–oxygen vibrations are typically observed at wavenumbers below 1000 cm^−1^ [26].

**Figure 5:**
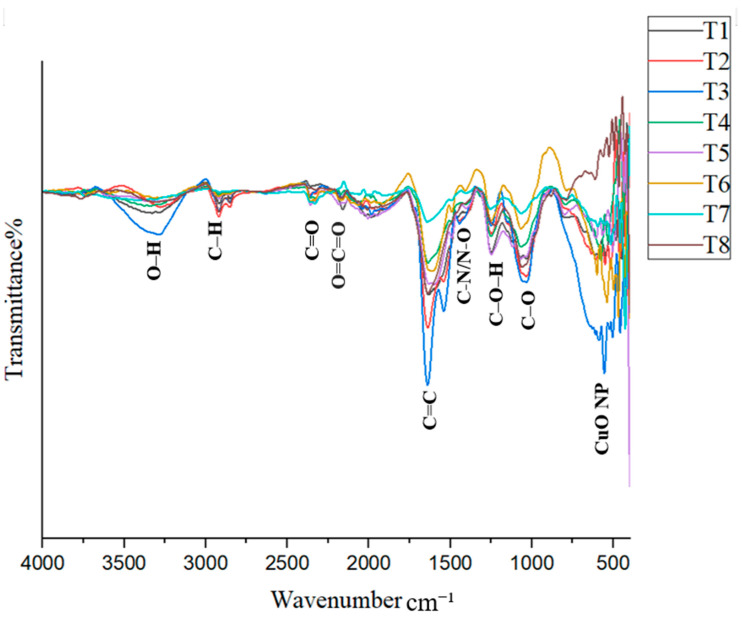
FTIR spectrum of CuO NPs in each treatment.

The formation of CuO NPs is indicated by peaks in the 500–650 cm^−1^ region. Ref. [19] reported a peak at 537 cm^−1^ corresponding to CuO NP formation, which was observed in treatment T4 in this study. Ref. [36] identified CuO NP-related peaks at 518.4 cm^−1^ and 600.1 cm^−1^, while [37] observed peaks at 522 cm^−1^ and 590 cm^−1^. Ref. [38] recorded a peak at 608 cm^−1^ associated with CuO NP formation. Ref. [23] also confirmed that peaks within the 400–600 cm^−1^ range can generally be attributed to CuO NPs. The consistent presence of peaks in the 500–650 cm^−1^ range across all treatments provides strong evidence for the successful synthesis of CuO NPs in this study (Figure 5). From 600 to 400 cm^−1^, each treatment exhibited unique peaks, further supporting CuO NP formation.

According to FTIR analysis, a broad peak observed in the range of 3228–3324 cm^−1^ is attributed to O–H stretching. A peak indicating C–H stretching from carboxylic acids or alcohols [23,37,39] was observed in all treatments except T7, within the range of 2916–2923 cm^−1^. A peak between 2323 and 2362 cm^−1^ was identified and attributed to C=O stretching in alkanes [24]. Additionally, a peak observed between 2156 and 2172 cm^−1^ indicated O=C=O stretching [38], and this was present in all treatments except T7. Another peak was recorded in the range of 1914–1984 cm^−1^ across treatments.

Furthermore, a characteristic peak between 1623 and 1647 cm^−1^, corresponding to C=C stretching [38], was found in all treatments. A peak in the range of 1323–1394 cm^−1^ was observed in treatments T1, T3, T4, T5, and T7. Ref. [24] reported a peak at 1399 cm^−1^, attributed to N–O bending, while [37] reported a peak at 1368 cm^−1^ associated with C–N stretching vibrations, indicating the presence of nitrogen compounds.

In the fingerprint region, a peak between 1241 and 1262 cm^−1^ was attributed to C–O–H stretching, while a peak between 1043 and 1067 cm^−1^ was assigned to C–N stretching in amines [40]. In treatments T5–T8, a peak in the range of 785–837 cm^−1^ was observed, which could be attributed to either C–Cl stretching or C=C stretching. Ref. [23] observed a peak at 834 cm^−1^ attributed to aromatic group bending vibrations. Ref. [26] reported that a peak at 2921 cm^−1^ corresponds to C–H stretching in alkanes, while a peak at 1043 cm^−1^ was linked to phenolic groups. Ref. [36] reported peaks associated with C–O bending at 1021.14 cm^−1^, C–H bending at 800.58 cm^−1^, O–H bending at 1412.3 cm^−1^, and C=C stretching at 1636.4 cm^−1^.

Interpreting FTIR spectra of nanoparticles can be challenging due to overlapping peaks, which complicates the identification of specific functional groups. Different researchers may assign the same peak to different groups, even when analyzing similar materials. This makes it difficult to correctly identify the functional groups responsible for nanoparticle synthesis and stabilization. In particular, signals from N–H and C–N bonds are often confused with those from O–H and C=O groups, as they appear in similar regions of the spectrum [40]. However, the results of this study confirm that all treatments successfully synthesized CuO nanoparticles.

### 3.5. UV–Visible Spectrophotometer Analysis

**Figure 6:**
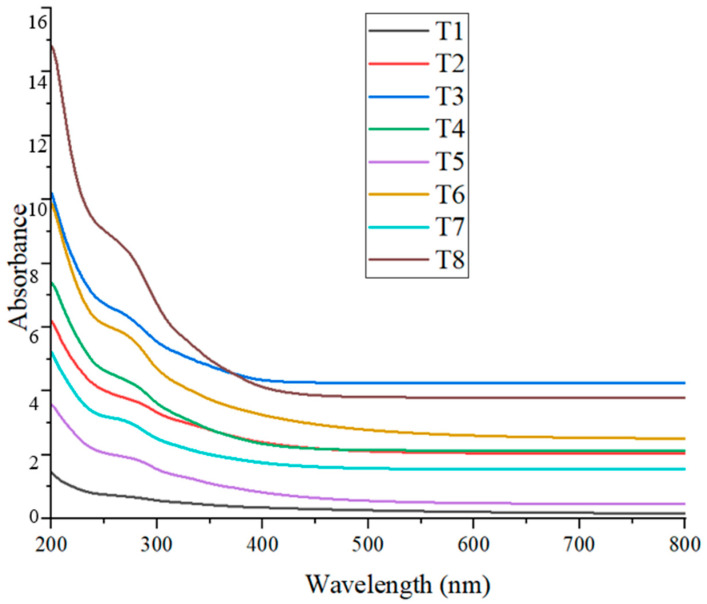
UV–Visible absorption spectra of CuO NPs synthesized under different treatments.

The presence of CuO nanoparticles in the colloidal solution was confirmed through UV–Visible spectrophotometry. The absorption peaks of the synthesized CuO NPs in this study ranged from approximately 265 nm to 285 nm (Figure 6), with slight variations observed across different treatments (Table 4). These values fall within the expected range for CuO nanoparticles, indicating successful nanoparticle formation.

Refs. [8,41] reported maximum absorbance for CuO NPs at approximately 282 nm, which closely aligns with the T2 (~285 nm) result from the present study. These researchers used *Suaeda maritima* (L.) Dumort and *Punica granatum* for nanoparticle synthesis, both yielding similar optical values, suggesting that plant-derived phytochemicals may consistently influence the surface plasmon resonance (SPR) of CuO NPs. Further studies by [19,42], who employed coffee extract and *Alchornea cordifolia* in the green synthesis of CuO NPs, reported absorbance peaks at 262 nm and 259 nm, respectively. These values are slightly shifted compared to those observed in the present study at T3 (~265 nm) and T7 (~267 nm), indicating minor differences likely due to the nature and concentration of phytochemicals present in the respective plant extracts.

In contrast, ref. [43] documented an absorbance peak at 400 nm after 100 min of synthesis, suggesting a possible shift caused by longer reaction times, particle aggregation, or differing reaction kinetics. The relatively lower wavelength values observed in the present study may reflect the early-stage formation of smaller or less aggregated CuO nanoparticles. Overall, the UV–Vis results obtained in this study are consistent with previous findings and support the successful biosynthesis of CuO nanoparticles using plant-based extracts. The variations in absorbance peaks among treatments and across studies can be attributed to differences in phytochemical composition, synthesis duration, and particle size, all of which influence the optical properties of the nanoparticles.

### 3.6. Phytochemical Screening of Leaf Extract

**Figure 7:**
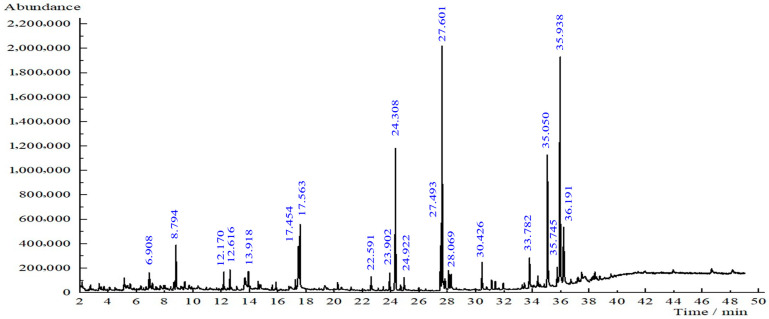
GC-MS chromatogram of *T. diversifolia* leaf extract.

Based on the GC-MS results, several compounds were detected in the *T. diversifolia* leaf extract (Figure 7). The most abundant constituents were phthalic acid (11.88% area at retention time (RT) 35.050 min), furfuryl hexyl ester (20.96% area at retention time (RT) 35.938 min), bis (2-ethylhexyl) phthalate (11.88%, RT 35.050 min), and 9-octadecenoic acid, methyl ester (16.52%, RT 27.601 min). Other notable compounds included hexadecanoic acid, methyl ester (9.56%, RT 24.308 min), pentane (5.92%, RT 17.454 min), 2,2,4-trimethyl-1,3-pentanediol diisobutyrate (4.44%, RT 17.563 min), neophytadiene (0.97%, RT 22.591 min), 4-vinylbenzene-1,2-diol (1.07%, RT 13.918 min), 9,12-octadecadienoic acid (1.34%, RT 28.237 min), Tagitinin C isomers (2.73%, RT 33.782 min), and methyl stearate (1.88%, RT 28.069 min). These compounds belong to a broad range of phytochemical classes, including fatty acid esters, terpenoids, esters, and phenolic derivatives. Ref. [14] also confirmed the availability of phytochemicals according to their GC-MS study.

Although differences in composition may exist between ethanolic and aqueous extracts, the ethanolic analysis provides useful preliminary insight into the phytochemicals present in the leaves that are potentially responsible for nanoparticle formation. Future work will involve more targeted phytochemical characterization of the aqueous extract to directly correlate specific compounds with their roles in the reduction and stabilization processes during nanoparticle synthesis.

The presence of phytochemicals can act as reducing, stabilizing, and capping agents in the green synthesis of copper oxide (CuO) nanoparticles. Their functional groups, such as hydroxyl (–OH), methoxy (–OCH_3_), and carbonyl (C=O), may participate in the reduction of Cu^2+^ ions to elemental copper (Cu^0^) and the subsequent formation of CuO [44]. Several studies have reported the role of phytochemicals in nanoparticle synthesis. According to [18], the mechanism involves an initial complexation of Cu^2+^ ions with phytochemicals, forming a complex with the plant extract. This complex undergoes hydrolysis and dehydration upon heating, finally yielding CuO nanoparticles. Similarly, ref. [45] described that phytochemicals in the extract reduce Cu^2+^ to Cu^0^ and simultaneously facilitate its oxidation to CuO, thereby completing the synthesis pathway. Thus, the GC-MS analysis confirms that *T. diversifolia* extract possesses the necessary biochemical compounds to enable the green synthesis of CuO nanoparticles. While these findings demonstrate promising synthesis conditions, further purification and functional validation, such as antimicrobial or cytotoxicity assays, are required to substantiate the nanoparticles’ suitability for biomedical or agricultural applications.

## 4. Conclusions

SEM imaging revealed that the average particle size of the synthesized nanoparticles ranged from approximately 125 to 150 nm, while the particle size analyzer reported slightly larger sizes. This discrepancy is attributed to the dynamic light scattering (DLS) method, which measures the hydrodynamic diameter, encompassing both the nanoparticle core and the surrounding organic capping layers. XRD analysis showed distinct crystalline peaks corresponding to CuO nanoparticles in treatments T1, T3, T4, and T8. Although FTIR and UV–Vis analyses confirmed CuO nanoparticle formation in the other treatments, these samples did not exhibit strong XRD peaks, possibly due to lower crystallinity or the presence of residual organic matter. Zeta potential measurements indicated that all treatments produced stable colloidal suspensions, with values ranging from −16.0 mV to −28.0 mV, suggesting good nanoparticle stability, which is beneficial for applications such as plant tissue culture. FTIR analysis further confirmed the presence of phytochemicals, contributing to several absorption peaks unrelated to CuO nanoparticles; however, peaks consistent with CuO were observed in the fingerprint region, aligning with previous studies. UV–Vis spectroscopic analysis preliminarily confirmed the formation of CuO nanoparticles in all treatments based on characteristic absorption peaks, with further analyses revealing additional distinguishing features. The results reveal the successful optimization of the green synthesis of CuO nanoparticles using *T. diversifolia* leaf extract. All treatments demonstrated the potential to produce CuO nanoparticles, albeit with varying efficiency and quality. The results suggest that lower concentrations of CuSO_4_, combined with higher reaction temperatures, are sufficient for effective nanoparticle synthesis. Considering the overall findings, treatment T4, characterized by a CuSO_4_ concentration of 5 mM, a reaction temperature of 80 °C, and a stirring time of 2 h, was identified as the optimal condition for CuO nanoparticle synthesis by *T. diversifolia*. Nevertheless, additional purification is recommended to enhance the purity and crystallinity of the final nanoparticle product. For future applications, particularly in biomedical or agricultural contexts, these additional purification steps will be essential to improve the overall quality of the nanoparticles.

## Figures and Tables

**Table 1:** Experimental conditions used for the green synthesis of CuO nanoparticles using *T. diversifolia* leaf extract.

Treatment	CuSO_4_ Concentration	Temperature	Stirring Time
T1	5 mM	60 °C	1 h
T2			2 h
T3		80 °C	1 h
T4			2 h
T5	10 mM	60 °C	1 h
T6			2 h
T7		80 °C	1 h
T8			2 h

**Table 2:** Particle sizes of CuO NPs in each treatment.

Treatment	Particle Size Range (nm)	Dominant Particle Size (nm)	Average Particle Size (nm)	Polydispersity Index (PDI)
T1	~100–800	~200	297.2 ± 63 ^d^	0.222
T2	~50–1000	~250	795.0 ± 79 ^b^	0.376
T3	~100–700	~150	683.9 ± 62 ^bc^	0.303
T4	~10–800	~300	362.3 ± 88 ^d^	0.380
T5	~100–1000	~400	1638.1 ± 43 ^a^	0.307
T6	~200–900	~500	592.0 ± 24 ^c^	0.473
T7	~300–1000	~600	592.5 ± 43 ^c^	0.348
T8	~50–900	~300	735.2 ± 16 ^bc^	0.335

Values marked with different letters are significantly different from each other (*p* < 0.05).

**Table 3:** Zeta potential values of CuO NPs in each treatment.

Treatment	Zeta Potential (mV)
T1	−16.43 ± 0.41 ^c^
T2	−19.50 ± 0.88 ^c^
T3	−20.40 ± 1.15 ^bc^
T4	−25.17 ± 4.51 ^ab^
T5	−16.13 ± 0.68 ^c^
T6	−18.03 ± 0.32 ^c^
T7	−20.96 ± 1.60 ^bc^
T8	−28.03 ± 2.05 ^a^

Values marked with different letters are significantly different from each other (*p* < 0.05).

**Table 4:** Absorption peak wavelengths of CuO NPs synthesized under different treatments.

Treatment	Wavelength
T1	~275 nm
T2	~285 nm
T3	~265 nm
T4	~280 nm
T5	~280 nm
T6	~270 nm
T7	~267 nm
T8	~273 nm

## Data Availability

The data presented in this study are available on request from the corresponding author.

## Abbreviations

The following abbreviations are used in this manuscript:

DLS	Dynamic Light Scattering
FTIR	Fourier-Transform Infrared
SEM	Scanning Electron Microscope
UV	Ultraviolet
XRD	X-ray Diffraction

## References

[1] Galpaya C., Induranga A., Vithanage V., Mantilaka P., Koswattage K.R. (2024). Comparative Study on the Thermal Properties of Engine Oils and Their Nanofluids Incorporating Fullerene-C_60_, TiO_2_ and Fe_2_O_3_ at Different Temperatures. Energies.

[2] Gunasena M., Alahakoon A., Polwaththa K., Galpaya G., Priyanjani H., Koswattage K.R., Senarath W. (2024). Transforming Plant Tissue Culture with Nanoparticles: A Review of Current Applications. Plant Nano Biol..

[3] Gunasena M., Galpaya G., Abeygunawardena C.J., Induranga D.K.A., Priyadarshana H.V.V., Millavithanachchi S.S., Bandara P., Koswattage K.R. (2025). Advancements in Bio-Nanotechnology: Green Synthesis and Emerging Applications of Bio-Nanoparticles. Nanomaterials.

[4] Induranga A., Galpaya C., Vithanage V., Indupama A., Maduwantha K., Gunawardana N., Wijesekara D., Amarasinghe P., Nilmalgoda H., Gunasena K. (2025). Nanofluids for Heat Transfer: Advances in Thermo-Physical Properties, Theoretical Insights, and Engineering Applications. Energies.

[5] Induranga A., Galpaya C., Vithanage V., Koswattage K.R. (2023). Thermal Properties of TiO_2_ Nanoparticle-Treated Transformer Oil and Coconut Oil. Energies.

[6] Altammar K.A. (2023). A Review on Nanoparticles: Characteristics, Synthesis, Applications, and Challenges. Front. Microbiol..

[7] Omran B.A., Omran B.A. (2020). Fundamentals of Nanotechnology and Nanobiotechnology. Nanobiotechnology: A Multidisciplinary Field of Science.

[8] Peddi P., Ptsrk P.R., Rani N.U., Tulasi S.L. (2021). Green Synthesis, Characterization, Antioxidant, Antibacterial, and Photocatalytic Activity of *Suaeda maritima* (L.) Dumort Aqueous Extract-Mediated Copper Oxide Nanoparticles. J. Genet. Eng. Biotechnol..

[9] Naika H.R., Lingaraju K., Manjunath K., Kumar D., Nagaraju G., Suresh D., Nagabhushana H. (2015). Green Synthesis of CuO Nanoparticles Using *Gloriosa superba* L. *Extr*. Their Antibact. Act. J. Taibah Univ. Sci..

[10] Dauthal P., Mukhopadhyay M. (2016). Noble Metal Nanoparticles: Plant-Mediated Synthesis, Mechanistic Aspects of Synthesis, and Applications. Ind. Eng. Chem. Res..

[11] Jadoun S., Arif R., Jangid N.K., Meena R.K. (2021). Green Synthesis of Nanoparticles Using Plant Extracts: A Review. Environ. Chem. Lett..

[12] Singh H., Desimone M.F., Pandya S., Jasani S., George N., Adnan M., Aldarhami A., Bazaid A.S., Alderhami S.A. (2023). Revisiting the Green Synthesis of Nanoparticles: Uncovering Influences of Plant Extracts as Reducing Agents for Enhanced Synthesis Efficiency and Its Biomedical Applications. Int. J. Nanomed..

[13] Mittal A.K., Chisti Y., Banerjee U.C. (2013). Synthesis of Metallic Nanoparticles Using Plant Extracts. Biotechnol. Adv..

[14] Roopa M.S., Shubharani R., Rhetso T., Sivaram V. (2020). Comparative Analysis of Phytochemical Constituents, Free Radical Scavenging Activity and GC-MS Analysis of Leaf and Flower Extract of *Tithonia diversifolia* (Hemsl.) A. Gray. Int. J. Pharm. Sci. Res..

[15] Robinson T.N., Justine D., Maxime P., Alexis S., Sidrine K.I., Hugues R.J., Ludovic C., Patrick M. (2024). Biological Activities and Phytochemical Constituents in the Stimulatory Potential of *Tithonia diversifolia* Fermented Extracts: A Review. Eur. J. Med. Plants.

[16] Tagne A.M., Marino F., Cosentino M. (2018). *Tithonia diversifolia* (Hemsl.) A. Gray as a Medicinal Plant: A Comprehensive Review of Its Ethnopharmacology, Phytochemistry, Pharmacotoxicology and Clinical Relevance. J. Ethnopharmacol..

[17] Oyetola E.O., Nwosu F.O., Oyeleke G.O., Olawale I.J., Mofoluwake Adegoke B., Afolabi U.O. (2023). Comparative Studies of Synthesized Copper Oxide Nanoparticles Using Aqueous Extract of Leaves. Nanochem. Res..

[18] Akintelu S.A., Folorunso A.S., Folorunso F.A., Oyebamiji A.K. (2020). Green Synthesis of Copper Oxide Nanoparticles for Biomedical Application and Environmental Remediation. Heliyon.

[19] Taghavi Fardood S., Ramazani A. (2016). Green Synthesis and Characterization of Copper Oxide Nanoparticles Using Coffee Powder Extract. J. Nanostruct..

[20] Millavithanachchi S., Gunasena M., Koswattage K., Kaluthanthri D., Bandara P., Galpaya G., Jambugaswaththa K., Kirthika V., Hansini A. Feasibility Study on the Green Synthesis of CuO Nanoparticles Using *Tithonia diversifolia* (Wild Sunflower) Leaf Extract. Proceedings of the International Conference on Emerging Technologies (ICET).

[21] Medjdoub A., Nedjimi M.S., Tlili S., Gherab K., Gherissi K. (2024). Green Synthesis of CuO Nanoparticles Using Aqueous Extract of (*Artemisia herba* Alba) and Their Potential Applications as Antimicrobial Agents. Pak. J. Life Soc. Sci..

[22] Khatamifar M., Fatemi S.J. (2022). Green Synthesis of Pure Copper Oxide Nanoparticles Using Quercus Infectoria Galls Extract, Thermal Behavior and Their Antimicrobial Effects. Part. Sci. Technol..

[23] Aroob S., Carabineiro S.A., Taj M.B., Bibi I., Raheel A., Javed T., Yahya R., Alelwani W., Verpoort F., Kamwilaisak K. (2023). Green Synthesis and Photocatalytic Dye Degradation Activity of CuO Nanoparticles. Catalysts.

[24] Dulta K., Koşarsoy Ağçeli G., Chauhan P., Jasrotia R., Chauhan P.K., Ighalo J.O. (2022). Multifunctional CuO Nanoparticles with Enhanced Photocatalytic Dye Degradation and Antibacterial Activity. Sustain. Environ. Res..

[25] Obayomi K.S., Oluwadiya A.E., Lau S.Y., Dada A.O., Akubuo-Casmir D., Adelani-Akande T.A., Bari A.F., Temidayo S.O., Rahman M.M. (2021). Biosynthesis of *Tithonia diversifolia* Leaf Mediated Zinc Oxide Nanoparticles Loaded with Flamboyant Pods (*Delonix regia*) for the Treatment of Methylene Blue Wastewater. Arab. J. Chem..

[26] Velsankar K., RM A.K., Preethi R., Muthulakshmi V., Sudhahar S. (2020). Green Synthesis of CuO Nanoparticles via Allium Sativum Extract and Its Characterizations on Antimicrobial, Antioxidant, Antilarvicidal Activities. J. Environ. Chem. Eng..

[27] Tamuly C., Saikia I., Hazarika M., Das M.R. (2014). Reduction of Aromatic Nitro Compounds Catalyzed by Biogenic CuO Nanoparticles. RSC Adv..

[28] Narayanan M., Srinivasan B., Sambantham M.T., Al-Keridis L.A., AL-mekhlafi F.A. (2022). Green Synthesizes and Characterization of Copper-Oxide Nanoparticles by *Thespesia populnea* against Skin-Infection Causing Microbes. J. King Saud Univ. Sci..

[29] Sarkar J., Chakraborty N., Chatterjee A., Bhattacharjee A., Dasgupta D., Acharya K. (2020). Green Synthesized Copper Oxide Nanoparticles Ameliorate Defence and Antioxidant Enzymes in *Lens culinaris*. Nanomaterials.

[30] Ahmed S., Ahmad M., Swami B.L., Ikram S. (2016). A Review on Plants Extract Mediated Synthesis of Silver Nanoparticles for Antimicrobial Applications: A Green Expertise. J. Adv. Res..

[31] Álvarez-Chimal R., García-Pérez V.I., Álvarez-Pérez M.A., Tavera-Hernández R., Reyes-Carmona L., Martínez-Hernández M., Arenas-Alatorre J.Á. (2022). Influence of the Particle Size on the Antibacterial Activity of Green Synthesized Zinc Oxide Nanoparticles Using *Dysphania ambrosioides* Extract, Supported by Molecular Docking Analysis. Arab. J. Chem..

[32] Thanh N.T., Maclean N., Mahiddine S. (2014). Mechanisms of Nucleation and Growth of Nanoparticles in Solution. Chem. Rev..

[33] Honary S., Barabadi H., Gharaei-Fathabad E., Naghibi F. (2013). Green Synthesis of Silver Nanoparticles Induced by the Fungus Penicillium Citrinum. Trop. J. Pharm. Res..

[34] Honary S., Barabadi H., Gharaei-Fathabad E., Naghibi F. (2012). Green Synthesis of Copper Oxide Nanoparticles Using *Penicillium aurantiogriseum*, *Penicillium citrinum* and *Penicillium waksmanii*. Dig. J. Nanomater. Bios..

[35] Dauthal P., Mukhopadhyay M. (2013). Biosynthesis of Palladium Nanoparticles Using *Delonix regia* Leaf Extract and Its Catalytic Activity for Nitro-Aromatics Hydrogenation. Ind. Eng. Chem. Res..

[36] Amin F., Fozia, Khattak B., Alotaibi A., Qasim M., Ahmad I., Ullah R., Bourhia M., Gul A., Zahoor S. (2021). Green Synthesis of Copper Oxide Nanoparticles Using *Aerva javanica* Leaf Extract and Their Characterization and Investigation of In Vitro Antimicrobial Potential and Cytotoxic Activities. Evid. Based Complement. Altern. Med..

[37] Nzilu D.M., Madivoli E.S., Makhanu D.S., Wanakai S.I., Kiprono G.K., Kareru P.G. (2023). Green Synthesis of Copper Oxide Nanoparticles and Its Efficiency in Degradation of Rifampicin Antibiotic. Sci. Rep..

[38] Singh D., Jain D., Rajpurohit D., Jat G., Kushwaha H.S., Singh A., Mohanty S.R., Al-Sadoon M.K., Zaman W., Upadhyay S.K. (2023). Bacteria Assisted Green Synthesis of Copper Oxide Nanoparticles and Their Potential Applications as Antimicrobial Agents and Plant Growth Stimulants. Front. Chem..

[39] Sukumar S., Rudrasenan A., Padmanabhan Nambiar D. (2020). Green-Synthesized Rice-Shaped Copper Oxide Nanoparticles Using Caesalpinia Bonducella Seed Extract and Their Applications. ACS Omega.

[40] Pasieczna-Patkowska S., Cichy M., Flieger J. (2025). Application of Fourier Transform Infrared (FTIR) Spectroscopy in Characterization of Green Synthesized Nanoparticles. Molecules.

[41] Ghidan A.Y., Al-Antary T.M., Awwad A.M. (2016). Green Synthesis of Copper Oxide Nanoparticles Using *Punica granatum* Peels Extract: Effect on Green Peach Aphid. Environ. Nanotechnol. Monit. Manag..

[42] Elemike E.E., Onwudiwe D.C., Singh M. (2020). Eco-Friendly Synthesis of Copper Oxide, Zinc Oxide and Copper Oxide–Zinc Oxide Nanocomposites, and Their Anticancer Applications. J. Inorg. Organomet. Polym. Mater..

[43] Veisi H., Karmakar B., Tamoradi T., Hemmati S., Hekmati M., Hamelian M. (2021). Biosynthesis of CuO Nanoparticles Using Aqueous Extract of Herbal Tea (*Stachys Lavandulifolia*) Flowers and Evaluation of Its Catalytic Activity. Sci. Rep..

[44] Yousaf Z., Saleh N. (2018). Advanced Concept of Green Synthesis of Metallic Nanoparticles by Reducing Phytochemicals. Nanobotany.

[45] Chakraborty N., Banerjee J., Chakraborty P., Banerjee A., Chanda S., Ray K., Acharya K., Sarkar J. (2022). Green Synthesis of Copper/Copper Oxide Nanoparticles and Their Applications: A Review. Green Chem. Lett. Rev..

